# Video-Rate Identification of High-Capacity Low-Cost Tags in the Terahertz Domain

**DOI:** 10.3390/s21113692

**Published:** 2021-05-26

**Authors:** Florent Bonnefoy, Maxime Bernier, Etienne Perret, Nicolas Barbot, Romain Siragusa, David Hely, Eiji Kato, Frederic Garet

**Affiliations:** 1 IMEP-LAHC Laboratory, Savoie Mont Blanc University, CNRS, Grenoble Alpes University, Grenoble INP, Scientific Campus, 73370 Le Bourget du Lac, France; florent.bonnefoy@univ-smb.fr (F.B.); maxime.bernier@univ-smb.fr (M.B.); 2 LCIS Laboratory, Grenoble INP, Grenoble Alpes University, ESISAR, 50 Rue Barthélémy de Laffemas, 26000 Valence, France; etienne.perret@lcis.grenoble-inp.fr (E.P.); nicolas.barbot@lcis.grenoble-inp.fr (N.B.); romain.siragusa@lcis.grenoble-inp.fr (R.S.); david.hely@lcis.grenoble-inp.fr (D.H.); 3 Advantest America, Inc., Princeton, NJ 08540, USA; eiji.kato@advantest.com

**Keywords:** low-cost and large capacity tag, 1D photonic band gap structure, Terahertz Identification

## Abstract

In this article, we report on video-rate identification of very low-cost tags in the terahertz (THz) domain. Contrary to barcodes, Radio Frequency Identification (RFID) tags, or even chipless RFID tags, operate in the Ultra-Wide Band (UWB). These THz labels are not based on a planar surface pattern but are instead embedded, thus hidden, in the volume of the product to identify. The tag is entirely made of dielectric materials and is based on a 1D photonic bandgap structure, made of a quasi-periodic stack of two different polyethylene-based materials presenting different refractive indices. The thickness of each layer is of the order of the THz wavelength, leading to an overall tag thickness in the millimetre range. More particularly, we show in this article that the binary information coded within these tags can be rapidly and reliably identified using a commercial terahertz Time Domain Spectroscopy (THz-TDS) system as a reader. More precisely, a bit error rate smaller than 1% is experimentally reached for a reading duration as short as a few tens of milliseconds on an 8 bits (~40 bits/cm^2^) THID tag. The performance limits of such a tag structure are explored in terms of both dielectric material properties (losses) and angular acceptance. Finally, realistic coding capacities of about 60 bits (~300 bits/cm^2^) can be envisaged with such tags.

## 1. Introduction

The idea to extend the concept of chipless RFID to the THz domain is mainly motivated by the necessity of fighting against counterfeiting, and thereby to reach higher security level for identification systems. Indeed, the devices of the most common tagging technologies (RFID tags and barcodes) can be easily counterfeited by reverse engineering [1,2,3] most of time because they are composed of metal parts or of printed schemes at the tag surface i.e., easily identifiable and could then be cloned in the case of high added value applications. Moreover, conventional RFID tags exhibit large dimensions (several centimetres), which depend directly on the corresponding RF wavelengths. The idea is to take advantage of both the optical and the radiofrequency (RF) domains i.e., the possibility to propose small device patterns to encode the information (by using shorter wavelengths than RF) and to keep these patterns out of sight by burying them under the surface of the tags (the main dielectric material is transparent enough in *THz* range, contrary to optical domain). On other hand, in case of chipless RFID, coding capacity remains much smaller than when integrating a chip. Chipless RFID is mainly based on resonator technology and the tag’s performance of course depends on the resonators design, number, the selected frequency range, and on the coding technique. Moreover, in the RF domain mainly, the design of chipless structures, and more generally the research for efficient performance, remains a hot topic subject with very rapid development [4,5,6,7,8]. The best performances published in the last two years reach about 30 bits but remains for the majority around 20 bits, corresponding to spatial density in the range of 1 bit/cm^2^, very exceptionally around 10 bits/cm^2^ or higher.

In contrast, the Terahertz Identification (THID) development is in its early stages and is currently limited by the development of the THz technologies, not only in terms of cost and compactness, but also in terms of the speed–reliability compromise of the reader, which is crucial for the intended applications. THID approaches have been initiated in 2003 by D. Cumming [9] who designed a THz hologram embedded in a dielectric material, which was opaque in the visible range but transparent in the THz one. In 2011, we proposed to encode binary information in the spectral response transmitted or reflected by tags, based on 1D quasi-periodic multilayer structures [10,11]. The information is thus contained in the volume of the tag, which can be directly embedded in the product to identify or its package. Thereby, the stealth of the tag does not make the latter visible at first glance. In real-word scenarios, its position should be either located by a specific design at the surface, or even kept secret as an additional level of protection, with a reading that would not be visible to everyone. This device principle was taken up in 2013 to encode binary information in the time domain by using the echoes of the incident wave reflected at the interfaces of the tag [12].

More recently, THID has attracted more pronounced attention due to advances in THz technologies, coupled to the evident possibilities to develop breakthrough solutions in order to push the limits of currently used technologies. In 2015, Y. Guan et al. [13] demonstrated an adaptation of the barcode for the THz range, using successive layers of polyethylene. In 2018, a 2D photonic-crystal slab matrix made of silicon was proposed as large-capacity tag, using both frequency and spatial domains [14]. Even if an interesting capacity of 48 bits/cm^2^ has been demonstrated, the device made of a periodic array of holes in a silicon slab remains unachievable by current low-cost industrial manufacturing techniques. In 2019, Cai Y. et al. [15] proposed a theoretical THID tag structure based on square graphene loops deposited on dielectric Topas material. A 2-bits Graphene Terahertz Identification Tag (GTIDT), with geometrical dimensions of several micrometers was first showed. Moreover, multilayer GTIDTs were investigated to form 3-bits structures. In 2019, we also demonstrated the principle to use a multilayer device including random inclusions in order to be used for THID identification, by using THz imaging associated with the Shift Invariant Wavelet Packet Decomposition (SIWPD) analysis method [16]. Finally, we also proposed a novel structure of device dedicating to identification and authentication, based on a diffractive grating engraved on a low-cost substrate [17]. By using different statistical analyses (Principal Component Analysis-PCA and Linear Discriminant Analysis-LDA), we show that the frequency signature of such a tag can be authenticated with a success rate of 99.994%. More recently, in 2020, Mitsuhashi et al. proposed a solution for real-time identification by combining a multi-wavelength injection-seeded THz parametric oscillator and a convolutional neural network algorithm [18]. A 46 bits’ tag has been demonstrated by combining 3 different materials in etalon type cavities of different thicknesses and arranged in a 2D array. At the same time, M. I. W. Khan et al. proposed a patch antenna array (2 × 2), built in a CMOS, to be used for identification at 260 GHz [19]. Even if this tag, made of a metallic pattern with a very small size of 1.6 mm^2^ is not “chipless” and remains out of the context of this study, it well illustrates the necessity to increase the useful frequency in order to reduce the size of the structure.

On the other hand, this study is also positioned in the perspective of very rapid developments that affect the size of potential technical solutions to read such tags in the THz range. Indeed, a low cost and compact solution have been proposed by M. Koch et al. [20] in 2015. This THz-TDS system uses cheap and commercially available components, such as for example, those taken from a DVD drive. In order to reduce the cost of the THz-TDS system, it is possible to replace the femtosecond laser by a cheaper and more classical Fabry–Perot laser diode [21]. Even if the performance of such THz-TDS systems is reduced, especially in terms of bandwidth, it remains sufficient for tag reading. Moreover, such an evolution can also be observed on continuous wave systems (THz-CW), which do not require a femtosecond laser. Such a spectrometer, based on DFB (Distributed Feedback Laser), can be of course less expensive and more compact, for example to be carried on a drone [22]. Some ongoing developments are also very encouraging to continue to reduce their cost and size, for example, by using multimode laser integrated on glass to be used for THz generation by frequency mixing [23].

In this context, we show that the prospect to operationally read and reliably identify a low-cost and easy to manufacture tag, is now made possible using commercially available THz spectrometers. We based our study on the structure we proposed in 2011 [10], fabricated using polyethylene to make it low-cost and compatible with industrial manufacturing techniques [11,24]. In this study, we experimentally show the possibility to read such an 8-bits tag in a quasi-real-time duration (<64 ms), with a rapid commercial Terahertz Time Domain Spectrometer (THz-TDS), while keeping a bit error rate smaller than 1%. We deduced from these results a criterion to ensure minimum reading reliability, that takes into account the reader performances, such as, for example, its signal to noise ratio. We also theoretically explore the impact of the losses of the material used to fabricate the tag on the coding capacity. Thus we show that the performances of such a tag can realistically extend up to about 60 bits by only using materials exhibiting an absorption coefficient of about 1 cm^−1^. Taking into account the size of the tag, much smaller than in the radiofrequency range, it corresponds to a surface density of about 300 bits/cm^2^, almost one order of magnitude higher than demonstrated in [14]. Finally, we experimentally evaluate that angular acceptance inversely decreases with the coding capacity, but tends to a limit of 5 degrees, despite the coding capacity from 20 bits. Let’s noticed that such angular acceptance remains sufficient for application using, for example, smart cards type objects.

## 2. Description of the THID Tag and of the Measurement Setup

The simplest form of the proposed THID tags is an 1D periodic stack of thin dielectric slabs, made of two different and transparent enough materials exhibiting high (*H*) and low (*L*) refractive index, respectively. On such a multilayer structure, an incident electromagnetic wave is either transmitted or reflected according to its frequency. The non-transmitted bandwidths are known as Photonic Band Gaps (PBGs) and appear periodically in the frequency domain. The spectral positions of these PBGs depend on the optical thicknesses of the quarter-wave equivalent layers, whereas their depth, also called rejection rate, depends on both the refractive index contrast between the successive layers H and L, and their number. When the optical thickness (refractive index multiplied by thickness) of at least one layer of the structure is modified (creation of a structural defect), the period of the stack is broken, altering in turn the transmitted spectral response of the structure: peaks appear in the PBGs. Both the number and frequency positions of these peaks are directly related to the structural defect characteristics [25]. Thereby, a given structural defect configuration leads to a unique THz transmitted (respectively reflected) spectral response which can be used as a signature to reliably identify the corresponding tag.

**Figure 1:**
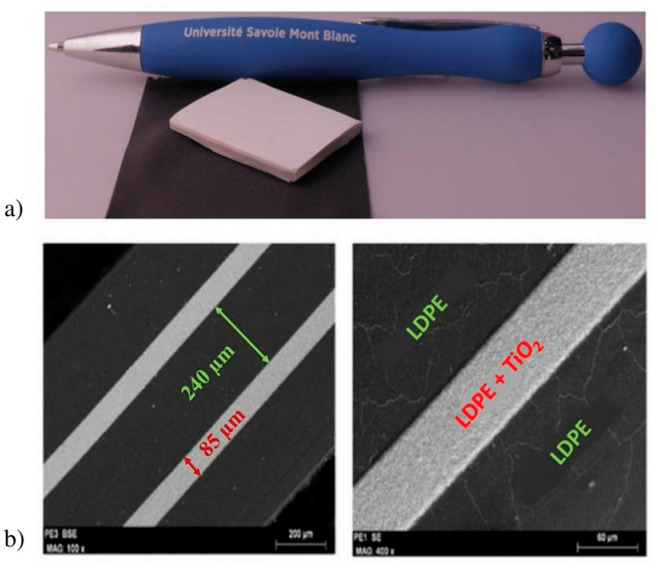
(**a**) Picture of the realized multilayer tag; (**b**) microscope images of stacked layers made of pure LDPE and TiO_2_ + LDPE mixture. © 2021 IEEE: this figure is plotted from [24].

**Figure 2:**
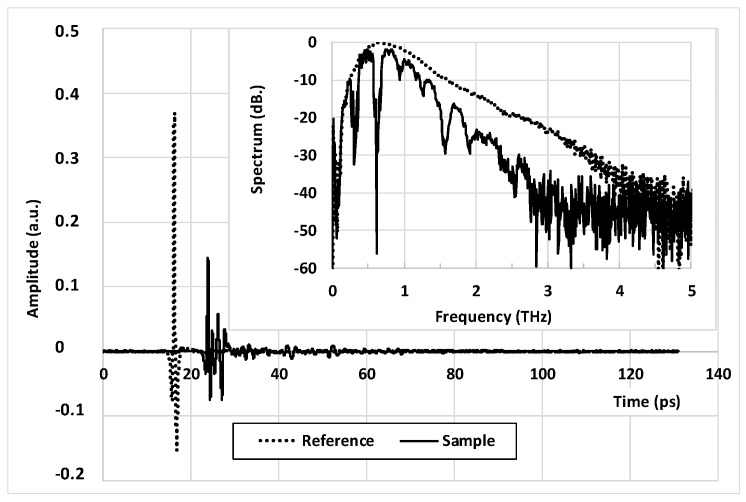
THz pulse delivered by the THz-TDS setup TAS 7500 SP System from ADVANTEST (dashed line) and its associated spectrum (inset). Solid lines correspond to the THz pulse transmit-ted through the developed multilayer THID tag and its associated spectrum (inset).

In order to obtain a high index contrast and, consequently, deep PBGs, we used pure Low-Density Polyethylene (LDPE) as *L* layer (*n_L_* = 1.51) and a mixture of TiO_2_ (60%)—LDPE (40%), as *H* one (*n_H_* = 2.29). The thicknesses of these layers (*e_L_* = 240 µm for *L* and *e_H_* = 85 µm for *H* as shown in Figure 1) were chosen to centre the first PBG around 310 GHz, where the THz reading systems present a high dynamic range (see Figure 2-inset). The number of layers (19) has been practically optimized (i) to maximize the signal rejection in the PBG, (ii) to limit the overall losses due to the absorption, and (iii) to keep the structure thickness in the millimetre range (see Figure 1a). Note that the whole thickness of the above described device remains reasonable (few millimetres) when working in the THz frequency domain, compared to the same type of device in *RF* (several tens of centimetres).

The setup used to characterize the tag is a commercial THz Time Domain Spectrometer (THz-TDS) system from ADVANTEST^®^ (model TAS7500 SP), which uses photoconductive antennas as the THz emitter and receiver. Contrarily to conventional THz-TDS systems based on mechanical delay lines [26], the pump/probe delay of the TAS7500 SP is achieved using an electrically controlled optical sampling (ECOPS) technique [27] that permits very high-speed measurements. This ECOPS-based system is then able to record a 132-ps long waveform in only 8 ms, with a time resolution of 2 fs. The spectral bandwidth spread typically from 0.1 to 4 THz (see Figure 2-inset) with a spectral resolution of about 7.6 GHz, whereas the signal to noise ratio and the dynamics depends, of course, directly on the number of waveforms averaged (from 1 to 4096).

The signature of the tag i.e., its complex transmission coefficient $\tilde{T}\left(\omega \right)$ of the tag is obtained by dividing the Fast Fourier Transform (FFT) of two THz waveforms (see Figure 2), the first one measured with the tag in between the antennas *S*(*t*), and the second one without *R*(*t*). $\tilde{T}\left(\omega \right)$ can be then written:

(1) $$\tilde{T}\left(\omega \right)=\frac{\tilde{S}\left(\omega \right)}{\tilde{R}\left(\omega \right)}$$

where $\tilde{S}\left(\omega \right)$ and $\tilde{R}\left(\omega \right)$ are the complex Fourier transform of the THz waveforms of *S*(*t*) and *R*(*t*), respectively.

**Figure 3:**
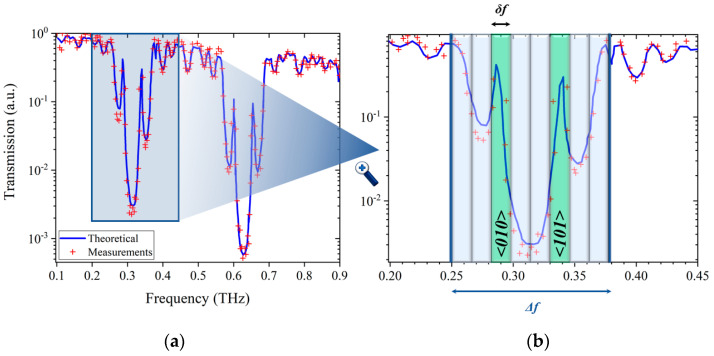
(**a**) Experimental (blue circles) and calculated (black continuous line) transmission spec-tra of the tag presented in Figure 1b. Focused on the first PBG whose limits are defined by blue vertical lines. Vertical strips represent the frequency coding channels. © 2021 IEEE: Figure (**b**) is plotted from [24].

For example, Figure 3 exhibits the frequency-dependent transmission coefficient of the tag previously described, in which a structural defect has been created by modifying the thickness of the central layer made of pure LDPE: *e_d_* = 1180 µm, instead of 240 µm. As previously pointed out, some peaks (two in the present case) of transmitted energy, occur in the first PBG. Then, we propose to use the presence of such transmitted peaks at some given frequencies to encode binary information, as described in the next section.

We can also notice that the modulus of the THz transmission of the tag can be perfectly predicted by the theory (see black continuous line) calculating by using a transfer matrix method [28]. The theoretical expression of $\tilde{T}\left(\omega \right)$ is then given by:

(2) $$T\left(\omega \right)=\frac{1}{\left|P_{11}\right|}$$

where *P*_11_ is the upper left element of the transfer matrix *P* that links the incident (*E_i_*), and reflected (*E_r_*) THz electric fields, with the transmitted one (*E_t_*):

(3) $$\left(\begin{array}{c} E_{i}\\ E_{r} \end{array}\right)=\left(\begin{array}{cc} P_{11} & P_{12}\\ P_{21} & P_{22} \end{array}\right)\left(\begin{array}{c} E_{t}\\ 0 \end{array}\right)$$

considering the above-described tag, and assuming an incoming THz beam under normal incidence, the transfer matrix writes:

(4) $$P=\underset{M}{\underbrace{A.S^{4}.\Delta }}.{\left(M^{*}\right)}^{-1}$$

M* is the complex conjugate of *M*, this latter being calculated from:

(5) $$A=\frac{1}{1+r_{1}}\left(\begin{array}{cc} e^{-i\frac{\omega }{c}n_{H}e_{H}} & r_{1}e^{i\frac{\omega }{c}n_{H}e_{H}}\\ r_{1}e^{-i\frac{\omega }{c}n_{H}e_{H}} & e^{i\frac{\omega }{c}n_{H}e_{H}} \end{array}\right)$$

(6) $$\Delta =\frac{1}{1+r_{2}}\left(\begin{array}{cc} e^{-i\frac{\omega }{c}n_{L}\frac{e_{d}}{2}} & r_{2}e^{i\frac{\omega }{c}n_{L}\frac{e_{d}}{2}}\\ r_{2}e^{-i\frac{\omega }{c}n_{L}\frac{e_{d}}{2}} & e^{i\frac{\omega }{c}n_{L}\frac{e_{d}}{2}} \end{array}\right)$$

(7) $$S=\;\frac{1}{1-r_{2}^{2}}\left(\begin{array}{cc} e^{-i\frac{\omega }{c}n_{L}e_{L}} & r_{2}e^{i\frac{\omega }{c}n_{L}e_{L}}\\ r_{2}e^{-i\frac{\omega }{c}n_{L}e_{L}} & e^{i\frac{\omega }{c}n_{L}e_{L}} \end{array}\right)\cdot \left(\begin{array}{cc} e^{-i\frac{\omega }{c}n_{H}e_{H}} & -r_{2}e^{i\frac{\omega }{c}n_{H}e_{H}}\\ -r_{2}e^{-i\frac{\omega }{c}n_{H}e_{H}} & e^{i\frac{\omega }{c}n_{H}e_{H}} \end{array}\right)$$

where $r_{1}=\frac{1-n_{H}}{1+n_{H}}$ and $r_{2}=\frac{n_{H}-n_{L}}{n_{H}+n_{L}}$ are the reflection coefficients at interfaces air/*H* and *H*/*L*, respectively.

## 3. Principle and Theory of Coding

To encode the binary information from the THz signature, obtained either in transmission or reflection, the PBG is divided into channels, each of them being labelled by a binary word. The transmitted peaks induced by the structural defect are then used to activate some of these channels (peak = channel activated, no peak = channel not activated). The corresponding binary code for the tag is then given by sorting and concatenating the binary codes of each activated channels. For example, in the case of Figure 3b, the PBG is divided in *N* = 8 channels, each of them corresponding to a 3-bits binary word. Channel 1 (at the lower frequency) and channel 8 (at the higher frequency) are associated to <000> and <111>, respectively. In the present case, the structural defect leads to *M* = 2 peaks that activate the 3rd and the 6th channels, each of them corresponding to <010> and <101>, respectively. Thereby, the corresponding binary word embedded into the spectral response can be coded over *k* = 6 bits: <010101>. Even though the obtained binary code is composed of 6 digits, the effective number of possible binary words cannot be practically equal to 2^6^ = 64, since some combinations are not allowed in this simple way of encoding. Indeed, the information is coded via the presence of peaks in given channels, without considering their magnitudes. Consequently, the system is not able to detect the presence of two superimposed peaks in the same channel. This means that, for example the code <010 010>, as well as for all the other “symmetric” ones, are not allowed. Moreover, as the activated channels are arbitrarily read from the left-hand side to the right-hand side, it implies that if the word <010101> can be obtained, the word <101010> cannot. Taking into account these limitations, the number of different codes achievable with the above-described coding technique is given by the combinations $C_{N}^{M}$ obtained by activating *M* channels among the *N* available, with *M* varying from 0 to = *N*. In turn, the total coding capacity *ρ* (in bits) of the presented tags is:

(8) $$\rho \left(N\right)=log_{2}\left(\overset{N}{\underset{M=0}{\sum }}C_{N}^{M}\right)=N$$

Thereby, the coding capacity (in bit) is equal to the number of channels *N* defined in the PBG, this latter depending directly on the PBG bandwidth ∆*f*, and the width of each channel *δf* (see Figure 3b):

(9) $$N=\left|\frac{\Delta f}{\delta f}\right|$$

If Δ*f* is fixed by the tag structure, *δf* can be limited by both the reader performance (spectral resolution) and the spectral bandwidth of the peaks. Indeed, to be distinguished, two peaks must be i. spectrally resolved and ii. separated by at least the full width at half maximum (FWHM) of the considered peaks whose value increases with the losses of the materials that constitute the layers in the tag. These two conditions constrain the channel width and in turn the coding capacity of the tag. Considering the frequency resolution of our reading system (about 7.6 GHz), which is limited by the maximum available time window to record the signal. Taking into account a value of ∆*f* = 130 GHz and a *δf* ~ 16 GHz (two time the spectral resolution to be sure to really identify a peak), it leads to a coding capacity of 8 bits. It corresponds to a spatial coding density of about 40 bits/cm^2^, as the THz beam diameter is about 5 mm.

**Figure 4:**
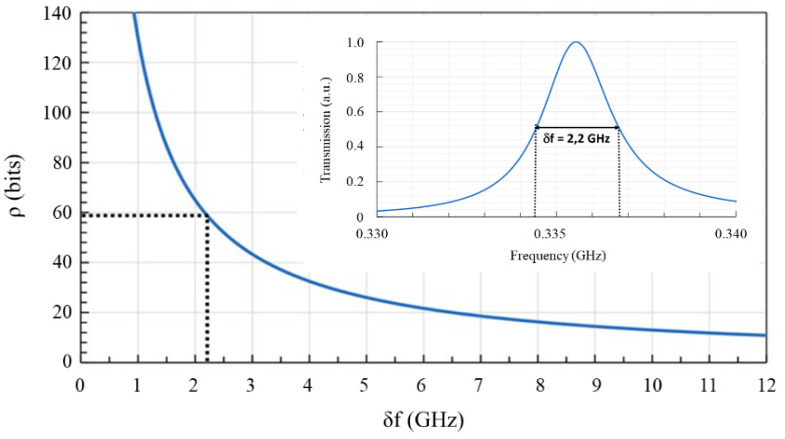
Prediction of the coding capacity of THID tags versus the channel width *δf* and considering a PBG bandwidth of ∆*f* = 130 GHz centered at 320 GHz. Inset: focus of the second peak in the 1st PBG, simulated by taking into account material absorption of 1 cm^−1^.

Nevertheless, to better evaluate the effect of these limiting parameters, we plot in Figure 4 the prospective evolution of the coding capacity *ρ* versus *δf*, keeping constant the other characteristics of the tag presented previously similar to the width of the first PBG (∆*f* = 130 GHz). Thus, the channel number *N* varies inversely with *δf*.

Note that the coding capacity *ρ* can reach several tens of bits, when *δf* goes below 3 GHz typically. In one hand, if such frequency resolution is hardly reached with classical TDS systems, it can be easily obtained using continuous wave (CW) systems whose frequency resolution goes typically down to 100 MHz. In that case, *δf* is no more limited by the reader but only by the FWHM of the transmitted peaks (for a given number of layers), i.e., by the layers’ absorptions. On other hand, considering realistic dielectric materials with absorption of about 1 cm^−1^ around 0.3 THz, we can estimate, by using the transfer matrix method, that the peaks width in such a structure would present a FWHM of 2.2 GHz (see insert of Figure 4). According to Figure 4, an eligible coding capacity of 60 bits can be then obtained, leading to theoretical spatial coding density of about 300 bits/cm^2^. Such a performance is several orders of magnitude better than coding density obtained with a chipless RFID system [4,5,6,7,8], mainly since THz wavelengths are a hundred times smaller than in the *RF*.

**Figure 5:**
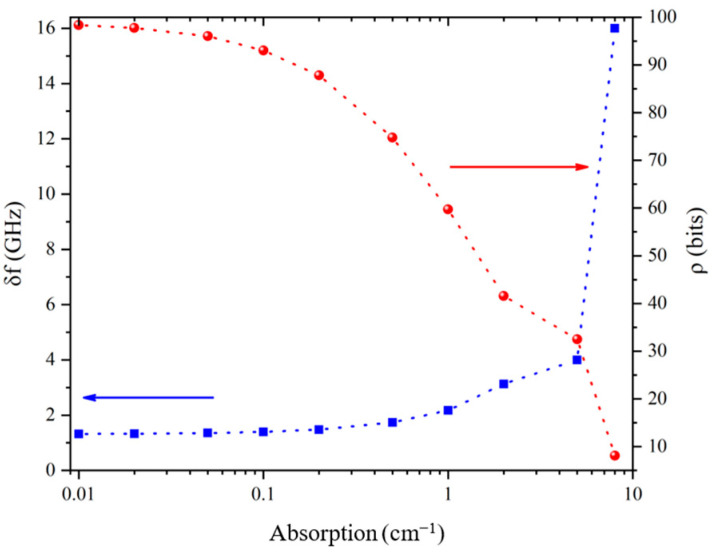
Variation of the channel width *δf* (in blue) and of the corresponding coding capacity *ρ* (in red) versus the absorption coefficient of the materials used to fabricate the 19 layers’ tag.

In order to analyse the effect, and consequently the performance limitations by the material used to fabricate the tag, we plot in Figure 5 the variation of the minimum channel bandwidth *δf* (blue curve) and then the corresponding coding capacity *ρ* (red curve) versus the absorption coefficient of the materials. We can easily see that *ρ* rapidly drops for absorption coefficient from 0.1 to 10 cm^−1^. However, as many dielectric materials exhibit low to moderate absorption in the frequency range typically below 1 THz, a coding capacity bigger than 30 bits can easily be considered with common plastic materials. Let’s noticed that these theoretical results have been obtained by taking into account the 19 layers’ structures described previously. Obviously, for a structure made of fewer layers, as long as this number remains sufficient to maintain an exploitable PBG, these results can be considered as underestimated.

Even though THID tags represent a potentially high coding capacity solution for identification applications, they will be of interest only if they are able to be reliably and rapidly read. Practically, THz signals recorded from the THz detector remain small; it follows that the corresponding signal to noise ratio drastically depends on the averaging rate, i.e., to the global acquisition time. Thereby, it exists as a speed–reliability compromise, which is discussed in the following section, where we focus on the evolution of reading reliability via the bit error rate (BER), when measurement duration is decreased down to allow video-rate reading.

## 4. Video Rate Identification

In this section we proceed to evaluate the ability of a current commercial THz system to precisely and rapidly get the signature of the THID tag described previously. To ensure a usable tag reading, the SNR of the chosen THz system must be large enough in the considered bandwidth, i.e., from 0.25 THz to 0.38 THz in the present case. Otherwise, the commercial TDS-THz systems that are currently available permit very fast acquisition of the frequency-dependent transmission coefficient of any sample under test, over a bandwidth spreading from 0.1 to 4 THz with a spectral resolution of several GHz. Such systems, as well as CW ones, can be obviously considered as good candidates for video-rate identification application, especially since their SNR remain sufficient. However, as the noise level is getting higher when measurement averaging is getting lower, the identification process becomes more and more hazardous as the acquisition time decreases. Therefore, we address below the problem to determine the highest reading speed compatible to a reliable identification process of the tag signature.

**Figure 6:**
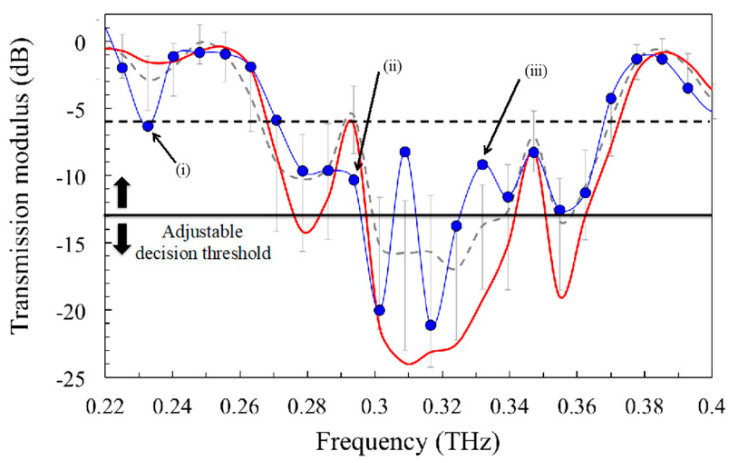
Example of a tag signature obtained using an acquisition time of 8 ms (blue dots and dashed curve), compared to the reference one (red continuous curve). The grey dashed curve is the transfer function obtained by averaging one hundred 8-ms measurements (error bars are given by the standard deviation). The horizontal lines represent the decision thresholds used to automat-tically define the PBG bandwidth limits (dashed line), and the peaks presence (solid line).

To estimate the shortest acquisition duration required to accurately identify the tag when read by the TAS7500 SP system, we compared one hundred spectral signatures measured on the 19-layer tag with the reference signature. For each set of measurements, we vary the number of averaging and thereby the whole measurement acquisition time, whereas the reference signature has been obtained using the largest averaging number (32,768), leading to an acquisition time of a few minutes (red curve in Figure 6).

Then, all the binary codes obtained by using the coding technique previously described on all of these signatures, are calculated and compared to the reference one: <010101>. Finally, the identification reliability is quantified by calculating the BER, which is simply obtained by counting the erroneous binary codes among the hundred measurements of each set. For all these steps, we used a homemade software based on a find-peak algorithm to objectively retrieve the binary code hidden in the signature. More precisely, the algorithm consists in two main steps:

- First, the spectral position and width of the PBG is determined by scanning the whole spectral signature. The PBG bandwidth is therefore simply defined as the spectral region where the modulus of the transfer function is lower than a given threshold, empirically chosen at -6dB in the present study (see horizontal black dashed line in Figure 6);
- Secondly, peaks are detected within the PBG using a peak-find algorithm considering only transmission peaks (1) whose magnitudes are greater than a given “decision threshold” (see horizontal black continuous line in Figure 6), and (2) which have spectrally separated each other by at least one channel width. As previously mentioned, this latter condition ensures a reliable detection of close peaks by taking into account the spectral resolution of the THz reader and FWHM of the peaks.

As an example, Figure 6 illustrates these two steps in the case of an 8 ms acquisition time measurement (blue doted curve), compared to the reference one (red curve). It is easy to see that the longer the acquisition time, the smaller the noise, the clearer the peaks appear in the PBG, and the better the binary code can be extracted. For 8 ms measurement duration, the noise level (represented by the grey error bars) is large enough to induce erroneous binary code extraction. Such noise-induced errors are of three kinds: (i) a transmission value, which is theoretically out of PBG bandwidth, gets lower than the PBG threshold (horizontal black dashed line): the PBG bandwidth is badly estimated as much as the frequency assignment of the channels, (ii) a defect peak is not taken into account as it keeps below the decision threshold (horizontal black continuous line): a channel is then not activated whereas it should be and (iii) a “noise peak” is detected as its amplitude becomes higher than the decision threshold, leading to the activation of a non-expected channel. Of course, the impact of the noise can be optimized by adjusting the two thresholds, especially the decision one that directly controls the unwanted activation, or inactivation of channels by the noise.

**Figure 7:**
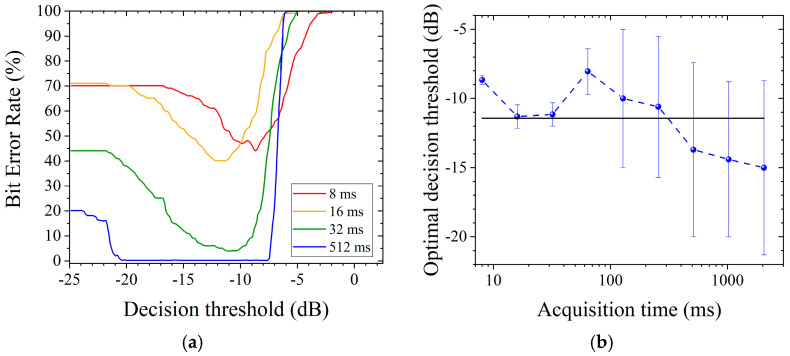
(**a**) BER versus the value of decision threshold, computed from different acquisition times. (**b**) Evolution of the decision threshold in function of the acquisition time for minimum BER (blue curve), and optimal decision threshold calculated from (black line).

In order to better understand the influence of the decision threshold, we plot in Figure 7a the behaviour of the BER versus this parameter for different acquisition times. First of all, Let noticed that the BER goes up to 100% when the decision threshold becomes higher than −8 dB, despite the acquisition duration. This threshold value corresponds to the magnitude of the peak cantered at 0.345 THz (see Figure 6). Indeed, in such a case, the peak can never be detected and thereby used to activate a channel, since the decision threshold is too high: the binary code cannot be retrieved and the BER tends to 100%. This behaviour is less drastic with short acquisition time since additional noise can potentially activate the expected channel, even if the decision threshold is higher than the peak magnitude (as illustrated by case (iii)) in Figure 6.

**Figure 8:**
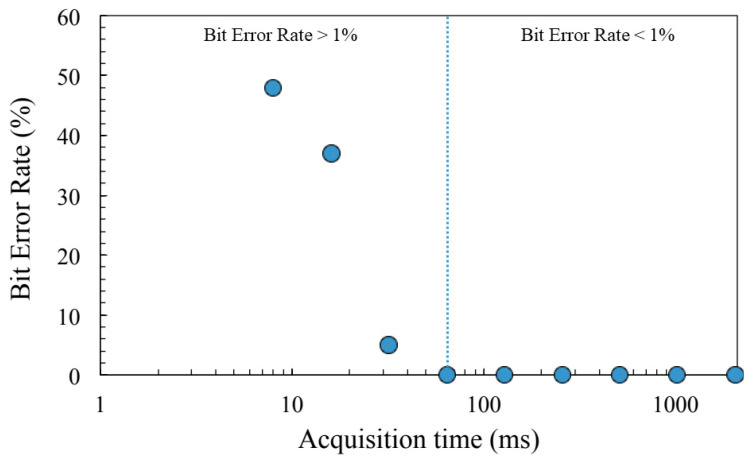
BER (%) calculated by using the 100 signatures of the tag, versus the acquisition duration.

On the other hand, when the decision is chosen lower, BER decreases to 0%, corresponding to an effective reliability of a reading smaller than 1%, before increasing again. This is obviously explained by the fact that the find-peak algorithm is more robust to the noise induced errors of kind ii and iii as long as the threshold decision remains in between the noise level and peaks magnitudes. Therefore, for each measurement time duration there is an optimal decision threshold position to obtain a minimum BER. We can notice in Figure 7b that this optimal threshold is roughly constant around −11 dB, despite the acquisition time. Note that this optimal threshold is then located 3 dB below the magnitude of the lowest peak (here cantered at 0.345 THz). This decision threshold value can be consequently used to determine the best value of the BER for all the different acquisition duration (Figure 8).

As expected, the BER becomes greater when acquisition time decreases. Considering the TAS7500 SP system, it appears that the tag is accurately identified as long as the acquisition time remains longer than 64 ms. In this case, the effective BER drops down below 1%, which corresponds to the minimum achievable limit of this study, as each set of measurements is based on one hundred tag signatures. Obviously, such a result depends on the other intrinsic performances of the THz system (dynamics, bandwidth, and spectral resolution), but also on the tag response (magnitude of the defect peaks, rejection level and bandwidth of the PBG).

**Figure 9:**
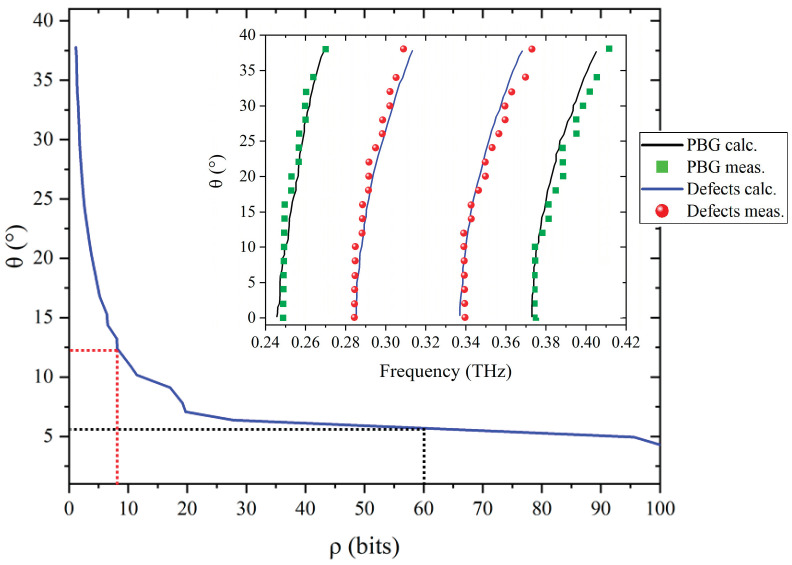
Maximum incident angle *θ* of the THz reading beam for the previous considered THID tag versus the coding capacity *ρ*. Insert: calculations (continuous lines) and measurements (dots) of the frequency shift of the PBG and defect peaks versus the incident angle of measurement.

Moreover, the electromagnetic response of such a multi-layered structure, i.e., the PBG width and peak positions, also closely depend on the incident angle of measurement *θ* (see insert of Figure 9). Indeed, under non normal incidence, the effective optical thickness of each layer is then increased, tending to a high frequency shift of the spectral signature. This angle-dependent behaviour can directly impact of the BER, by the activation of unwanted channels and leading to a wrong corresponding binary code. To compensate for such a frequency-shift impact, we must increase the channels width *δf*. According to Expressions (8) and (9), the number *N* of channel decreases, as well as the coding capacity. In order to fix a limit acceptance angle, we plot in Figure 9 the impact of the incident angle on the ultimate coding capacity based on the tag structure previously described. Considering the first analysis, the initial coding capacity of 8-bits remains unchanged since the incidence angle remains smaller than 12°. However, in the case of a larger capacity around 60 dB, the angular acceptance drops down to 5°. Let noticed that for a coding capacity from about 25 bits, such angular acceptance keeps roughly constant.

## 5. Conclusions

In this work, we have experimentally demonstrated the ability to identify an 8-bit coding capacity (coding density ~40 bits/cm^2^) THID tag only made of dielectric materials and by using a commercial THz-TDS spectrometer. The tag signature is read with a reliability rate bigger than 99 percent in only a few tens of milliseconds. Such a performance has been obtained by comparing the binary words encoded in the frequency domain by the spectral position of two defect modes appearing in the first photonic band gap of a few-mm thick tag, constituted of 19 layers of LDPE and of LDPE-TiO_2_ mixture (40/60 volume ratio), alternately stacked. To ensure such success rate, we define a decision threshold (~11 dB) as an absolute criterion to reliably, find the frequency position of the peaks, and therefore, the binary code of the tag. Consequently, it implies that a THz reading system must exhibit a sufficiently large SNR to perform both video-rate and reliable reading of a tag (>18 dB). Let noticed that the SNR of course depends on the averaging rate but also on the intrinsic noise sources in THz-TDS systems [29]. The performance limits, in terms of coding capacity, have been also explored both versus the material permittivity (dielectric losses) and the angular acceptance.

On the other hand, the recent developments of ultrafast high-resolution ASOPS-based THz-TDS systems [30] or in the longer term, THz-CW systems [22], associated with de-noising extraction techniques [31,32], would provide a powerful THz reader, perfectly suited for the video-rate identification of tags exhibiting large storage capacities. Indeed, a better performance would be obtained by improving the frequency resolution of the reader, leading to a larger number of channels, and by increasing the number of peaks in the PBG from the tag side [33].

Based on these results, we showed that such a multilayer tag family is able, with the realistic improvements suggested previously on both the tag and the reader, to exhibit coding capacity up to 60 bits, corresponding to a coding density of about 300 bits/cm^2^, i.e., more than 6 times bigger than the one reported by Mitsuhashi et al. [14]. Moreover, the structures proposed in [14,18] are made of a 2D arrangement of several “unitary cells”, whose size of each of them is very close to that of our entire tag. The surface of these devices then increases with the coding capacity, while it remains constant in our case. Indeed, the surface of our tag is only limited by the THz beam diameter, itself limited by the diffraction phenomenon (in case of far field reading); it will then be smaller the higher the frequency.

Finally, the optimization of the technique used to encode the binary information, for example by taking into account either the Q-factor and/or the magnitude of each defect peaks, would also considerably increase the performances of the whole system.

## Data Availability

Not applicable.
